# Zoonotic Pathogens in Food: New Advances and Editorial Insights

**DOI:** 10.3390/foods14162847

**Published:** 2025-08-17

**Authors:** Maria Schirone, Mirella Luciani, Luigi Iannetti

**Affiliations:** 1 Department of Bioscience and Technology for Food, Agriculture and Environment, University of Teramo, 64100 Teramo, Italy or m.luciani@izs.it; 2 Istituto Zooprofilattico Sperimentale dell’Abruzzo e del Molise “G. Caporale”, 64100 Teramo, Italy; l.iannetti@izs.it

## 1. Introduction

This Editorial introduces the Special Issue “New Advances in Management and Characterization of Zoonotic Pathogens in Foodstuffs and Food Processing Facilities”, which brings together recent insights into the behavior, adaptation, detection, and control of zoonotic pathogens in food and food-related environments. The contributions collected in this Special Issue fall into three main thematic areas: microbial adaptation mechanisms, innovative diagnostic approaches, and novel antimicrobial strategies.

Infections transmitted through contaminated food remain a significant public health threat in both industrialized and developing nations, placing millions of individuals at risk each year [1]. It is estimated that the global burden of unsafe food consumption results in approximately 600 million cases of foodborne illness and 420,000 deaths annually, with children under the age of five accounting for nearly 30% of all fatalities [2]. These risks are exacerbated by poor hygiene practices, improper food storage, inconsistent regulatory enforcement, and infrastructural deficiencies, especially in low- and middle-income countries [3]. Moreover, the emergence of multidrug-resistant strains among foodborne pathogens poses an increasing challenge for public health and food safety, necessitating integrated approaches combining surveillance, antimicrobial stewardship, and consumer education [4]. Infections are primarily linked to pathogenic microorganisms such as *Salmonella* spp., *Listeria monocytogenes*, *Escherichia coli*, and *Campylobacter* spp. [5,6], while viral agents including Norovirus and Hepatitis A virus represent additional major threats to food safety worldwide [7]. Notably, several of these bacteria are zoonotic pathogens that pose significant risks to vulnerable populations, including the young (fetuses, infants, and toddlers), elderly, and immunocompromised individuals, for whom the illness can result in severe or even fatal outcomes [8]. Parasitic organisms including *Giardia lamblia*, *Taenia* spp., and *Entamoeba histolytica* are also widespread, particularly in areas with poor hygiene standards, ineffective food safety policies, and underdeveloped public health infrastructure [9,10]. Although less frequently reported, fungal species such as *Penicillium*, *Claviceps*, *Aspergillus*, and *Fusarium*—among the roughly 300 of an estimated 1.5 million fungi known to be pathogenic to humans—can contribute to food-related illnesses through the production of mycotoxins, representing a notable public health concern [11]. These epidemiological trends underscore the urgent need for improved detection methods, a deeper understanding of pathogen survival mechanisms, and the development of targeted interventions to reduce contamination risk throughout the food chain. In response to these challenges, the articles collected in this Special Issue explore complementary lines of research that offer new insights into pathogen detection and control.

## 2. Exploring New Frontiers in Foodborne Pathogens

### 2.1. Stress Response Mechanisms

The first group of contributions in this Special Issue focuses on the physiological and molecular adaptation of foodborne pathogens, particularly *L. monocytogenes*, under environmental and technological stress conditions. D’Onofrio et al. (contribution 1) investigated proteome modulation in *L*. *monocytogenes* ST7 when exposed to suboptimal conditions such as low pH, refrigeration temperatures, and high salt concentrations. By combining 1D electrophoresis, 2D-PAGE, and tandem mass spectrometry, the study identified 1160 proteins revealing condition-specific expression of key virulence factors such as Internalin A and Listeriolysin O. These findings underscore the bacterium’s remarkable ability to adapt and persist in hostile food environments. Importantly, the study showed that the presence of pathogenic proteins in the proteome—rather than the mere detection of their genes—is a more reliable indicator of potential virulence, especially under stress. The authors highlight that deep proteomic knowledge across strains is crucial, as stress responses and protein expression patterns vary depending on environmental conditions typical of food production and storage. Future directions include the bioinformatic characterization of additional proteins using predictive tools such as VirulentPred to assess virulence potential and Vaxijen v.2.0 to evaluate immunogenic potential associated with virulence pathways. Additionally, the development of CRISPR-Cas-assisted recombineering systems is planned to implement targeted gene edits. This would enable functional studies to confirm the pathogenic role of stress-induced proteins and potentially guide the development of novel control strategies or diagnostic markers.

Furthermore, an immunoproteome analysis of a *L*. *monocytogenes* ST7 strain exposed to mild acid and salt stress identified 226 proteins, including 58 immunogenic antigens, highlighting stress-induced expression of pathways implicated in oxidative stress, carbohydrate metabolism, and DNA repair. These results underscore the strain-specific nature of adaptation and pathogenic potential, supporting the value of integrating proteomic and transcriptomic data to pinpoint reliable virulence markers beyond genomic predictions [12].

Building on these findings, Szymczak (contribution 2) examined atypical *L*. *monocytogenes* (aLm) isolates obtained from soil, food, and swabs collected in food industry environments. These strains, characterized by the absence of hemolysis and motility, exhibited high phenotypic and genotypic diversity, including variable sugar fermentation profiles, multi-drug resistance, and the presence of only a subset of virulence genes. The study revealed that non-hemolytic aLM strains accounted for over 10% of all *L*. *monocytogenes* isolates—a markedly higher proportion than previously reported—and that their emergence appears to be influenced by environmental and processing factors in both animal- and plant-based food chains. The research emphasized the importance of phenotypic fingerprinting for distinguishing aLM from both classical *L*. *monocytogenes* and other *Listeria* species, noting that traits such as lack of hemolysis are strongly correlated with virulence gene profiles (e.g., *prfA*, *inlB*, and *mpl*). These isolates exhibited higher levels of resistance to antibiotics, including clindamycin, penicillin, streptomycin, and tetracycline, raising concerns about their potential as underrecognized reservoirs of resistance and pathogenicity. Given that standard ISO detection methods may fail to identify these strains, the study argues for the revision of current detection protocols and recommends further investigation using tools such as MALDI-TOF and genomic sequencing, especially to assess the risk of horizontal gene transfer to more virulent *L*. *monocytogenes* populations.

Parra-Flores et al. (contribution 3) complemented these findings by applying whole-genome sequencing (WGS) to 16 *L*. *monocytogenes* strains isolated from ready-to-eat refrigerated foods in Chile. The authors identified a high prevalence of sequence type ST5 and serotype 1/2b and found a consistent presence of virulence and stress response genes across strains. Furthermore, they identified resistance determinants such as *brcBC* and *qacJ* genes, plasmid elements, and mobile genetic elements associated with environmental persistence. Their study demonstrates the potential of WGS for accurate strain typing, improved surveillance, and enhanced food safety risk assessment, especially in cold-chain products. Importantly, the genomic analysis revealed resistance to antibiotics, heat stress, and disinfectants, suggesting that these ST5 strains have developed adaptive traits that may allow them to withstand industrial hygiene treatments, thereby contributing to their long-term persistence. The use of WGS was shown to provide high-resolution insights that surpass conventional typing methods, enabling more targeted and effective sanitization strategies. This study reinforces the idea that precise pathogen characterization is fundamental for designing evidence-based food safety interventions and protecting public health.

The persistence of *L*. *monocytogenes* in food processing environments is often linked to its ability to form biofilms on surfaces such as stainless steel, polystyrene, glass, and plastic, particularly where cleaning and sanitization are insufficient [13]. In these biofilms, bacterial cells are embedded within a self-produced matrix of extracellular DNA, proteins, and polysaccharides (notably teichoic acids), which enhances their resistance to disinfectants and environmental stresses [14,15]. The capacity for biofilm formation varies depending on factors such as temperature, bacterial strain, and the nature of the surface, influencing the pathogen’s ability to persist [16,17,18,19]. Flagella-driven motility significantly contributes to initial surface attachment and biofilm development, although *L*. *monocytogenes* can also adhere through flagella-independent mechanisms. Understanding these complex interactions is essential for improving control strategies in food industry settings. However, recent evidence suggests that the relationship between biofilm formation and persistence is more intricate than previously assumed, as WGS studies have yet to identify biofilm-specific markers consistently linked to persistence. This discrepancy may arise from varying definitions of persistence, diverse experimental designs, or strain-specific interactions. Studies mimicking realistic conditions, including mixed microbial communities and the presence of food residues, are crucial to unravel the mechanisms underlying *L*. *monocytogenes* survival and persistence in industrial environments. Such insights will support the design of effective interventions to reduce long-term contamination risks [20].

### 2.2. Advances in Detection Technologies

Understanding pathogen adaptation is crucial, but rapid and precise detection methods are equally essential for effective food safety management. In this regard, Counihan et al. (contribution 4) assessed the potential of long-read sequencing (Oxford Nanopore MinION) for the identification of Shiga toxin-producing *E. coli* (STEC) directly from ground beef samples. The method demonstrated the ability to identify key virulence genes within just a few hours in pure cultures and enriched food samples, offering promising prospects in reducing diagnostic time in routine food safety testing while requiring significantly less manual labor. After standard enrichment, DNA extraction, library preparation, and sequencing setup could be completed in under two hours, with informatic analysis taking less than thirty minutes. Besides pathogen identification, the approach also allows for serotyping and the detection of antimicrobial resistance genes, offering a comprehensive overview from a single assay. Moreover, the potential to detect multiple pathogens simultaneously within the same sequencing run suggests strong future applicability for routine surveillance. Although certain technical challenges remain—such as managing interference from host DNA—the findings position long-read sequencing as a fast, high-throughput, and information-rich alternative for food safety diagnostics, particularly in high-risk products such as ground beef.

These advances in pathogen detection reflect a broader trend toward the integration of omics sciences in food safety surveillance. Foodomics—a multidisciplinary field combining genomics, proteomics, metabolomics, and bioinformatics—is transforming our ability to assess food quality, traceability, and microbiological safety across the entire supply chain. Initially developed in biomedical research, omics technologies are now extensively applied to food analysis, enabling molecular-level detection of chemical residues, microbial metabolites, allergens, and emerging contaminants [21]. Among the various omics disciplines, genomics plays a crucial role in animal breeding programs and food product authentication, improving traceability and genetic quality [22,23,24]. Proteomics and metabolomics provide essential insights for optimizing the nutritional content, safety, and sensory properties of animal-derived products such as milk and meat [25,26], while lipidomics offers detailed fatty acid profiling to support the creation of functional foods with enhanced health benefits [27]. At the same time, increasing attention to sustainability, animal welfare, and transparency is driving the advancement of rapid, precise technologies for food quality evaluation, meeting both consumer demands and regulatory requirements [28].

Metabolomic techniques, including NMR and mass spectrometry, have been widely employed to characterize microbial spoilage, monitor bacterial inactivation and detect toxic compound formation in diverse food matrices such as fish, dairy products, honey, and wine. These high-precision methods enhance detection sensitivity and serve as early warning systems for contamination, thereby bolstering consumer protection and food system resilience [29]. Despite these advantages, challenges remain, including high implementation costs, the need for specialized infrastructure, and the complexity of integrating heterogenous, high-dimensional multi-omics datasets. Overcoming these obstacles requires advanced computational approaches such as machine learning and deep learning, which demand expertise that is still limited in many contexts [30].

### 2.3. Leveraging Nature for Food Safety

The third group of contributions in the Special Issue addresses innovative antimicrobial strategies based on natural compounds. Al-Kharousi et al. (contribution 5) evaluated the chemical composition and antimicrobial activity of essential oils and smoke derived from Hojari and Sha’bi grades of Omani frankincense (*Boswellia sacra*). Using GC-MS, the authors identified monoterpenes and sesquiterpenes as the primary constituents and demonstrated broad-spectrum efficacy against Gram-positive and Gram-negative bacteria, yeasts, and molds. Notably, both oil grades showed comparable antimicrobial profiles, attributable to their similar chemical makeup, suggesting interchangeability for use in food and medicinal applications, though further work is needed to validate specific use cases. Among tested microorganisms, *Saccharomyces cerevisiae* and *Fusarium solani* emerged as the most sensitive to the essential oils. The study also highlighted the potential of frankincense smoke as a natural sanitation agent: in some cases, complete microbial inhibition was observed, including against *Staphylococcus aureus* and *E*. *coli*, as well as airborne molds and yeasts. However, the detection of fine particulate matter in the smoke raises concerns regarding inhalation safety, especially for vulnerable individuals. Overall, the findings support the promising role of frankincense-derived products in natural food preservation and environmental hygiene, provided that their application is matched with appropriate safety assessments.

Finally, Sepúlveda et al. (contribution 6) investigated the antimicrobial efficacy of cinnamaldehyde and vanillin, both in their free and encapsulated forms, in a protein-enriched apple juice beverage. Encapsulation with whey protein concentrate markedly improved the antimicrobial performance of cinnamaldehyde, enabling significant reductions in *E*. *coli*, *Listeria innocua*, and *S. cerevisiae* populations, and underlining its potential as an additive in dairy-based drinks. In contrast, vanillin encapsulation did not enhance its antimicrobial capacity, underscoring the need for further research to develop more effective delivery systems for this compound. The study also determined that the co-application of both encapsulated compounds could yield additive effects against bacteria and synergistic inhibition of yeasts, suggesting formulation strategies to broaden the spectrum of antimicrobial activity. To analyze microbial inactivation patterns, the authors employed both the modified Gompertz and Weibull models: the Gompertz model provided detailed kinetic parameters useful for comparing free and encapsulated formulations, while the Weibull model offered insights into the distribution of microbial resistance levels. These findings underscore the relevance of encapsulation technologies in optimizing natural preservatives and the value of mathematical modeling in guiding shelf-life prediction and product design.

## 3. Conclusions

Collectively, the studies featured in this Special Issue offer a comprehensive and multifaceted overview of current challenges and emerging solutions in the management of zoonotic pathogens in the food sector. From deciphering microbial adaptation to advancing diagnostic technologies and exploring novel antimicrobial agents, each contribution emphasizes the importance of integrated, science-based approaches. Looking ahead, interdisciplinary collaboration among microbiologists, food technologists, bioinformaticians, and public health professionals will be essential to translate these insights into scalable, real-world interventions. Standardizing experimental protocols, improving pathogen detection in complex environments, and leveraging omics-based surveillance tools will be key to enhancing food safety frameworks globally. As zoonotic threats continue to evolve, sustained research and innovation will remain critical pillars in strengthening food system resilience and safeguarding public health. This Special Issue not only enriches current scientific understanding but also helps define the priorities for future investigations in food safety and zoonotic pathogen control.
